# Recurrent orbital schwannomas: clinical course and histopathologic correlation

**DOI:** 10.1186/1471-2415-12-44

**Published:** 2012-08-31

**Authors:** Michelle Kron, Brenda L Bohnsack, Steven M Archer, Jonathan B McHugh, Alon Kahana

**Affiliations:** 1 University of Michigan Medical School, Ann Arbor, MI, USA; 2 Department of Ophthalmology and Visual Sciences, University of Michigan, Ann Arbor, MI, USA; 3 Department of Pathology, University of Michigan, Ann Arbor, MI, USA; 4 Helmut F. Stern Career Development Professor, Ophthalmology and Visual Sciences, University of Michigan, Ann Arbor, MI, USA

## Abstract

**Background:**

Schwannomas are slow-growing typically encapsulated tumors composed of differentiated Schwann cells, the primary class of peripheral glial cells. Complete excision is the treatment of choice for orbital schwannomas that cause pain, disfigurement, diplopia, or optic neuropathy. The presence of multiple schwannomas in a single patient suggests possible association with neurofibromatosis type 2 (NF2) or schwannomatosis.

**Case presentation:**

We present 2 patients who experienced recurrent orbital schwannoma without evidence for neurofibromatosis. The recurrence in one patient, a 59-year old man, occurred 6 years after complete excision of the initial tumor. This recurrence consisted of 2 independent tumors in the same orbit. The recurrence in the second patient, a 5 year-old girl, occurred multiple times within days to weeks of partial excisions until eventually a complete excision was performed.

**Conclusion:**

The clinical history, histopathologic features and particularly the intraoperative findings suggest that the 59 year old man suffers from orbital schwannomatosis, while the rapid recurrence in the second patient correlated with the cellular features of her plexiform schwannoma. Hence, the recurrence in each patient is linked to a different etiology, with implications for treatment and patient counseling given the difficulty in treating orbital schwannomatosis. To our knowledge, this is the first description of isolated orbital schwannomatosis.

## Background

Schwannomas are slow-growing typically encapsulated tumors composed of differentiated Schwann cells, the primary class of peripheral glial cells. They can occur in any location, but occur infrequently in the orbit, representing only 1% of orbital tumors [1,2]. Schwannomas are most common in the 20- to 50-year-old age group, but may occur at any age. No frequency variation according to sex has been identified [3]. A review of the literature suggests that in the post-CT era, very few cases of pediatric schwannoma have been reported [4,5].

Complete excision is the treatment of choice for orbital schwannomas that cause pain, disfigurement, diplopia, or optic neuropathy [6-8]. Histologically, schwannomas are usually encapsulated with alternating cellular schwann-cell rich Antoni-A areas and less cellular and myxoid Antoni-B areas (reviewed in [9]), which immunohistochemically stain strongly for S-100 protein [10].

The presence of multiple schwannomas in a single patient suggests possible association with neurofibromatosis type 2 (NF2) or schwannomatosis. The hallmark of NF2 is bilateral vestibular schwannomas resulting from *NF2* tumor suppressor gene germline mutation. Schwannomatosis is characterized by multiple pathologically proven schwannomas without vestibular tumors diagnostic of NF2. Although most schwannomatosis cases are sporadic, instances of autosomal dominant transmission with incomplete penetrance have also been reported [11], though linkage analysis has now excluded the NF2 locus, suggesting schwannomatosis is a distinct entity [12-15].

Accurate characterization of schwannomatosis is important as it is a prognostic factor that helps guide treatment. Here we present two cases, a 59-year old man with clinically recurrent orbital schwannomatosis, manifesting as multiple independent schwannomas that likely arose *de novo*, contrasting with a 5-year old girl with a case of a recurrent isolated orbital schwannomas who underwent 2 surgical excisions with recurrence of the tumor within 1 month of each surgery.

## Case presentation

### Case 1

**Figure 1:**
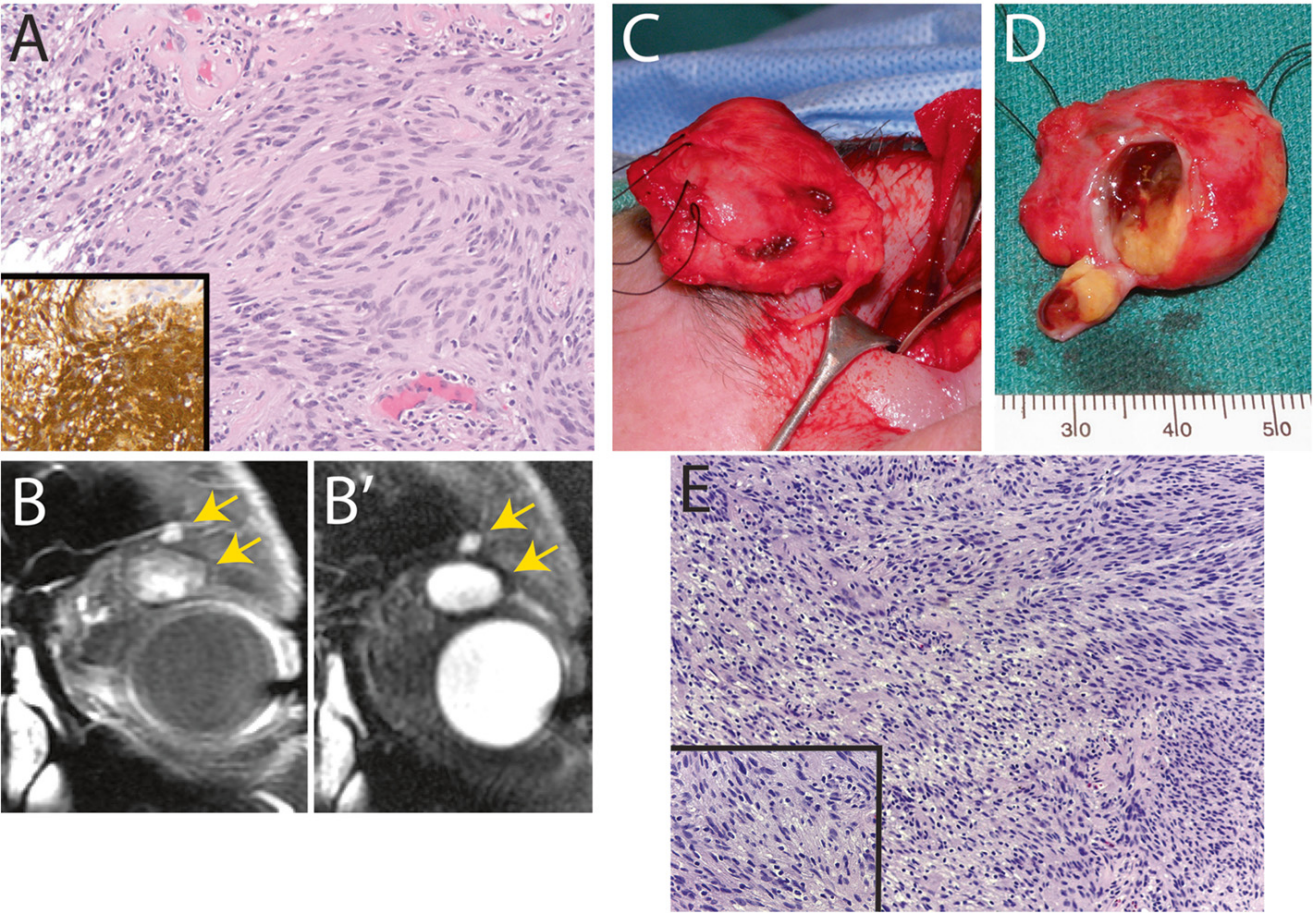
**Case 1. Orbital schwannomatosis. A**: Histopathology of first tumor excision (H&E and IHC) revealing S-100 positivity (inset), indicating schwannoma. Original magnification 200x. **B**: Magnetic resonance imaging of the recurrent tumor demonstrating two distinct well-circumscribed superior orbital lesions (arrows) with patchy enhancement on T1 (**B**) and T2 (**B’**) imaging. **C**: Intraoperative photo of excised mass. **D**: Gross pathology of excised mass, with window in capsule showing contents. E: Histopathology of recurrent lesion (H&E) revealing schwannoma. Original magnificantion 100x and 400x (inset).

A 59-year old man with a past medical and surgical history significant for diabetes mellitus, hypertension, sleep apnea, and thyroid lobectomy for goiter first presented in September 2004 complaining of intermittent vertical diplopia. On examination, there was a palpable mass of the superomedial orbit. An MRI demonstrated this to be a dense, well-circumscribed lesion, and a 27 x 10 x 10 mm mass was completely excised via a left anterior orbitotomy. Histopathologic exam revealed a schwannoma with intense positivity for S-100 protein (Figure 1A). Post-operatively, the diplopia improved but did not resolve, and he developed upper lid ptosis that was surgically repaired via levator plication in 2005.

The patient returned to our clinic in 2010, reporting increased diplopia, left-sided proptosis, left forehead hypesthesia and left upper lid droop. An MRI of the orbits revealed a large left superior orbital mass with patchy enhancement on T2 imaging (Figure 1B). The mass was noted to be located more laterally than the 2005 schwannoma location. He underwent left anterior orbitotomy with complete resection of a 27 x 25 x 15 mm orbital mass (Figure 1C, D), with partial sacrifice of superior oculomotor nerve fibers. In addition, a second 8 x 5 x 2 mm mass, distinct from the primary mass and located superiorly on the edge of the orbital rim was observed intraoperatively and excised completely. This second mass was clearly spatially distinct from the larger tumor (Figure 1B-B’, arrows).

Histopathology revealed both masses to be schwannomas with similar histopathologic patterns, including clusters of lipidized xanthoma cells (Figure 1E). Post-operatively, the proptosis resolved, but the patient continued to complain of diplopia and worsened ptosis, and underwent successful strabismus and frontalis sling surgeries. A complete neurologic and medical evaluation failed to reveal any additional clinical features that would suggest neurofibromatosis. The findings support the hypothesis that each schwannoma arose *de novo*, representing orbital schwannomatosis.

### Case 2

The second patient was a 5-year old girl who presented with a left medial canthal mass increasing in size over the previous 1 year. At that time the patient was undergoing chemotherapy for acute lymphocytic leukemia (ALL). She underwent excision of this mass at an outside facility with histopathology suggesting schwannoma. The mass recurred within 1 month of excision and the patient underwent a second excision 4 months after the first surgery, with pathology again suggesting schwannoma. Within 1 month of excision, the mass again began to enlarge and by 1 year had returned to its original size. At this point the patient was referred to our clinic for further evaluation and treatment.

At our initial evaluation, the patient’s ALL was in remission. She was otherwise healthy and developmentally normal without evidence of other abnormal masses or signs of neurofibromatosis. On ophthalmologic examination, the patient’s visual acuity was 20/20 bilaterally with normal pupil examination and extraocular motility. External examination revealed a mobile 9 x 10 mm left medial canthal mass resting on the underlying orbital bone with an overlying scar. The remainder of her examination was unremarkable. An MRI scan revealed a well-circumscribed, contrast enhancing mass without posterior extension and possible bone involvement. The pathology slides from the specimens obtained during the two previous excisions were reviewed, revealing plexiform cellular schwannoma.

**Figure 2:**
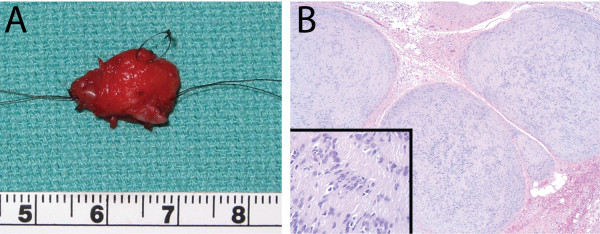
**Case 2. Plexiform cellular schwannoma. A**: Gross pathology of excised mass measuring 15 mm by 12 mm. **B**: Histopathology (H&E) revealing schwannoma. Original magnification 50x and 200x (inset).

The patient underwent left medial anterior orbitotomy with complete excision of the 15 x 11 x 5 mm canthal mass with uninvolved margins (Figure 2A). Histopathology again illustrated a plexiform arrangement with the lesion divided into bundles, each of which surrounded a remnant nerve, with characteristic nodularity, fascicles of palisading spindle cells with storiform appearance, and lack of necrosis or myxoid changes (Figure 2B). Immunohistochemical stains confirmed the morphologic diagnosis with diffuse S-100 protein positivity. Given the risks of radiation in a child and the lack of aggressive morphology, this treatment option was not pursued. At 3 and 6-month follow-up evaluations, there were no signs of recurrence. Further workup to assess for the possibility of neurofibromatosis was negative.

## Conclusions

Our cases demonstrate different mechanisms for schwannoma recurrence: residual tumor in the case of the 5-year old girl (leading to rapid recurrence), and likely *de novo* recurrence in the case of the 59-year old man (leading to late recurrence).

Although orbital schwannomatosis is a rare entity, our 59-year old male patient (case 1) fits previously proposed diagnostic criteria, with multiple independent histologically confirmed schwannomas in his left orbit and no evidence of NF2 [16,17]. Although the schwannomas had similar histopathological patterns and were in proximity to one another, they were in spatially distinct both surgically and radiologically (Figure 1 B/B’). Schwannomatosis can be heritable or sporadic, manifests as a propensity to develop schwannomas in the absence of the usual signs of NF2, and can be associated with any nerve in the orbit [6,18,19]. It is notable that the new schwannomas in this patient occurred years, rather than months, after the initial excision. However, it remains theoretically possible that the recurrent tumors in case 1 were seeded at the time of the original tumor resection in 2005.

Interestingly, at the time our 5-year old girl (case 2) was originally diagnosed with orbital schwannoma, she was undergoing chemotherapy treatment for ALL. There is one case in the Japanese literature of a patient diagnosed with a thoracic schwannoma while in remission from acute lymphoblastic leukemia, although with only two known cases, a causal relationship between ALL and schwannomas cannot be concluded [20]. Patients with NF1 are predisposed to juvenile myelomonocytic leukemia [16], but our patient had no other signs of NF1 despite a complete evaluation. Case 2 is somewhat unusual in that the isolated orbital schwannoma recurred twice, each within 1 month of excision. Despite the plexiform features, and although the previous excisions were most likely incomplete, it is still rare that the tumor recurred so rapidly. Malignant peripheral nerve sheath tumors are much more aggressive lesions and can quickly recur even with complete excision [21], but histopathology of the final specimen showed only benign features, and there were no further recurrences.

While surgery is the mainstay of treatment for orbital schwannomas, radiation therapy is an alternative treatment is some cases. However, even with vestibular schwannomas, the efficacy of radiation therapy is unclear [22]. Furthermore, radiation exposure carries significant risk for secondary malignancies [23], and hence the use of radiation therapy to treat benign conditions should be approached with caution.

In summary, we report 2 cases of recurrent orbital schwannomas, demonstrating the challenge of treating this tumor. In both cases, the patients should be carefully monitored for further recurrences. In cases of schwannomatosis, patients should be advised that the goal of therapy may be mostly palliative, and that close monitoring would be required in order to facilitate early intervention of further recurrences, before the tumor size becomes problematic. In the case of plexiform schwannomas, the patient and family should be advised of the risk of early recurrence.

## Consent

Written informed consent was obtained from the patient or patient’s guardian for publication of this case report and any accompanying images. A copy of the written consent is available for review by the Editor-in-Chief of this journal.

## Abbreviations

NF2: Neurofibromatosis type 2; ALL: Acute lymphocytic leukemia.

## Competing interests

The authors declare they have no competing interests.

## Authors’ contributions

MK, BB and AK drafted the manuscript. AK conceived of the case series, performed surgeries, and coordinated the specimen analysis. JM assisted in analysis of the pathology specimens and summary of histopathologic findings. SA performed surgery and assessed eye movement. All authors read and approved the final manuscript.

## Authors’ information

AK and SA are practicing ophthalmologists, specializing in oculoplastics and pediatrics, respectively. JM is a practicing pathologist. BB is a pediatric ophthalmology fellow and MK is a transitional year resident.

## Pre-publication history

The pre-publication history for this paper can be accessed here:

http://www.biomedcentral.com/1471-2415/12/44/prepub
